# Study of the Effect of Nanoparticles and Surface Morphology on Reverse Osmosis and Nanofiltration Membrane Productivity

**DOI:** 10.3390/membranes3030196

**Published:** 2013-08-15

**Authors:** Yuming Fang, Steven J. Duranceau

**Affiliations:** Department of Civil, Environmental and Construction Engineering, University of Central Florida, P.O. Box 162450, Orlando, FL 32816-2450, USA; E-Mail: yuming_fang@knights.ucf.edu

**Keywords:** productivity, nanoparticles, reverse osmosis, nanofiltration, cake growth model

## Abstract

To evaluate the significance of reverse osmosis (RO) and nanofiltration (NF) surface morphology on membrane performance, productivity experiments were conducted using flat-sheet membranes and three different nanoparticles, which included SiO_2_, TiO_2_ and CeO_2_. In this study, the productivity rate was markedly influenced by membrane surface morphology. Atomic force microscopy (AFM) analysis of membrane surfaces revealed that the higher productivity decline rates associated with polyamide RO membranes as compared to that of a cellulose acetate NF membrane was due to the inherent ridge-and-valley morphology of the active layer. The unique polyamide active layer morphology was directly related to the surface roughness, and was found to contribute to particle accumulation in the valleys causing a higher flux decline than in smoother membranes. Extended RO productivity experiments using laboratory grade water and diluted pretreated seawater were conducted to compare the effect that different nanoparticles had on membrane active layers. Membrane flux decline was not affected by particle type when the feed water was laboratory grade water. On the other hand, membrane productivity was affected by particle type when pretreated diluted seawater served as feed water. It was found that CeO_2_ addition resulted in the least observable flux decline, followed by SiO_2_ and TiO_2_. A productivity simulation was conducted by fitting the monitored flux data into a cake growth rate model, where the model was modified using a finite difference method to incorporate surface thickness variation into the analysis. The ratio of cake growth term (*k*_1_) and particle back diffusion term (*k*_2_) was compared in between different RO and NF membranes. Results indicated that *k*_2_ was less significant for surfaces that exhibited a higher roughness. It was concluded that the valley areas of thin-film membrane surfaces have the ability to capture particles, limiting particle back diffusion.

## 1. Introduction

In RO and NF membrane treatment processes, fouling is one of the major issues related to the deterioration in membrane performance. Efforts have been made to reduce membrane fouling by improving membrane properties, optimize operational conditions and advanced pretreatment of the feed water, however, fouling is still inevitable [1,2,3]. Colloidal fouling of membranes is caused by different mechanisms. For RO, NF, and perhaps some tight UF membranes, colloidal fouling is caused by the particles accumulating on the membrane surface to develop a so-called cake layer. This cake layer provides an additional hydraulic resistance to water permeating through the membrane, therefore reducing the water flux. For MF and UF membranes, pore plugging is another factor that causes membrane fouling besides the particle accumulation on the surface. The extent of pore plugging and cake formation depends on the relative size of the particles compared to the membrane pores sizes [4].

Particulate fouling has been shown to relate to the membrane surface roughness in RO and NF membrane processes in bench scale experiments [5,6,7]. Depending on the particle’s size, density, and membrane surface roughness, fouling may occur due to accumulation of particles on the membrane surface resulting in a build-up of the cake layer. An increase in particle concentration typically leads to an increase in fouling, while smaller particles either causes more, or less, fouling as compared to larger particles [8,9]. In addition, the ionic strength of the solution is an additional factor that can affect membrane fouling. As the ionic strength increases, the fouling potential increases as a result of the double layer compression formed around the colloids [10,11,12]. With the application of atomic force microscopy (AFM), membrane active-layer characteristic such as surface morphology, pore sizes, and surface porosity can be determined and correlated to membrane fouling behavior. The AFM images presented in the work of Vrijerhoek *et al*. depict membrane surfaces as having an elevated ridge and depressed valley morphology. They concluded that the fouling behavior was related to the degree of surface roughness [13].

To investigate the effects of chemical and physical interactions between the particles and the membranes, silica dioxide (SiO_2_), titanium dioxide (TiO_2_), and cerium dioxide (CeO_2_) served as foulants during the conduct of the fouling experiments. SiO_2_ is a stable metal oxide and is generally found in natural waters and has been identified as one of the possible foulants of synthetic membrane processes. A number of studies have been performed using SiO_2_ in membrane colloidal fouling experiments [5,7,9,10,11,12,13]. TiO_2_ is a well known photocatalyst, and exhibits properties of oxidative decomposition; the photo-induced ultrahydrophilicity of TiO_2_ has attracted much interest in both basic and applied sciences. TiO_2_ has a unique self-cleaning effect in that photocatalysis and hydrophilicity can take place simultaneously on the same surface even though the mechanisms are completely different [14]. By comparison, less research has been conducted on the study of CeO_2_ in membrane fouling studies. Cerium dioxide has been demonstrated as a self-cleaning catalyst with a strong absorption for ultraviolet radiation but having a lower photocatalytic activity for visible light. In general nanoparticles have been used to modify membrane surface properties in order to enhance membrane performance and mitigate membrane fouling [15,16]. The use of nanoparticles in membrane manufacturing allows for both a high degree of fouling control and the ability to produce a desirable membrane structure. Some researchers have tried to synthesize membranes with titanium oxide (TiO_2_) nanoparticles either trapped inside or deposited on the surface to modify the membrane surface roughness and hydrophobicity [15,17]. In this study, silica, titanium and cerium nanoparticles were applied to RO and NF membranes in a cross flow flat sheet test unit. The flux decline for RO and NF membranes was then monitored so that comparisons between the different nanoparticles could be accomplished.

The conventional filtration theory for flow through porous media is known as Darcy’s law [18]. Considering resistance in series, a fouling model was established by applying a resistance value to each component of membrane fouling. Note that each component contributes to hydraulic resistance and that they act independently from one another. Typical forms of the resistance-in-series model is shown in Equation (1). The pore constriction resistance coefficient *κ_p_* can be negligible for small pore membranes such as NF and RO.

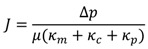
(1)
where:

*κ_m_*, *κ_c_*, *κ_m_* = Resistance coefficient for membrane, cake layer, and pore constriction;

*µ* = Dynamic viscosity.

This fundamental fouling model has been modified by Hoek [19] by considering the effect of enhanced osmotic pressure. According to this modified model, the osmotic pressure at the RO membrane active layer tends to be enhanced when a cake layer has been formed, depending on cake thickness and concentrate fluid salinity. This phenomenon can be accounted for by incorporating the osmotic pressure difference *∆π^∗^_m_* in the basic filtration model, as follows:

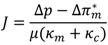
(2)

With a constant trans-membrane pressure (TMP), several filtration models have been developed to describe the fouling processes [20]. These models relates the permeate flow (*Q*), permeate volume (*V*), the time (*t*) with the filtration constants for each model (*K_b_, K_i_, K_s_, K_c_*), and the initial permeate flow (*Q*_0_). The mathematical expressions of these models and their assumptions are shown in Table 1 [20]. Among these models, the intermediate blocking filtration and the cake filtration models are applicable for RO and NF membranes. The remaining models are applicable to UF and MF membranes.

**Table 1:** Constant pressure filtration model.

Model	Equation	Assumption
Complete blocking filtration	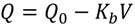	Particles are not superimposed on one another, the blocked surface area is proportional to the permeate volume
Intermediate blocking filtration	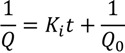	Particles can overlap each other, not every deposited particle block the pores
Standard blocking filtration	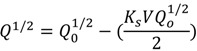	Particles are small enough to enter the pores, the decrease of pore volume is proportional to the permeate volume
Cake filtration	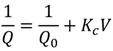	Particles are big enough to not enter the pores, and therefore forms a cake layer on the surface

The applications of these models can be seen in many publications. Mohammadi and his coworkers studied the flux decline in RO processes during separation of oil-water emulsions, where it was observed that the best fitting was the intermediate blocking filtration model [20]. Lim investigated the fouling behavior of microfiltration membranes in activated sludge system [21]. The results show that the main types of membrane fouling in this case were attributed to initial pore blocking (standard blocking filtration model) followed by cake formation (cake filtration model). Bolton compared these four models in application to MF and UF of biological fluids, where the combination of a cake filtration model with a complete pore blocking model resulted in the best fit of experimental data [22]. Alternatively, in a cross flow UF experiment conducted by Tarabara [8], cake formation was investigated under variable particle size and solution ionic strength. It was found that a dense layer of the colloidal deposit formed adjacent to the membrane with an abrupt transition to a much more porous layer near the membrane-suspension interface [8]. These studies from MF and UF implied that different models should be considered at different phases when simulating fouling behavior of RO and NF. Other effort has been explored to investigate the effects of pressure, membrane rejection, fluid shear, and the effect of cake-enhanced concentration polarization on the fouling behavior of different membranes. It was concluded pressure, rejection and fluid shear are important in determining the cake deposition under most testing conditions [23].

The objective of this study is to investigate the productivity of RO and NF membranes under laboratory-scale conditions. It is postulated that nanoparticles impact flux decline depending on membrane morphology. This paper reports on the results of an investigation conducted to investigate the intrinsic mechanism of nanoparticle interaction with surface properties on the productivity of membranes in aqueous environments.

## 2. Experimental Design

### 2.1. Preparation of Membrane and Nanoparticles

It has been found that surface chemical heterogeneities can provide favorable sites for attachment onto what is otherwise an unfavorable surface for colloid adherence [24]. To take into account different membrane surface properties, both RO and NF membrane sheets having different surface roughness were purchased for study (Sterlitech, Kent, WA, USA). The specifications of the membranes investigated in this study are shown in Table 2. The membrane samples were acquired as dry sheets and were stored in distilled (DI) water at room temperature prior to assembly into flat-sheet test cells. The membranes were characterized for intrinsic physical and chemical properties through the use of surface roughness and contact angle.

**Table 2:** Specification of membranes used in the experiments.

Designation	Membrane type	Manufacturer	Polymer	* MWCO	Pressure, psi
BW30	RO	Dow	Polyamide	100D	260
XLE	RO	Dow	Polyamide	100D	130
CK	NF	GE Osmonics	Cellulose Acetate	2000	220

* MWCO: Molecular Weight Cut Off.

Commercial TiO_2_ (anatase, 99%, 15 nm), CeO_2_ (99.9%, 50–105 nm), and SiO_2_ (99+%, 80 nm) nanoparticles (NanoAmor, Houston, TX, USA) were used in the fouling experiments. The nanoparticles were supplied in powder forms. The true densities were 2.2–2.9 g/cm^3^ for SiO_2_, 3.9 g/cm^3^ for TiO_2_, and 7.1 g/cm^3^ for CeO_2_. This size range is to ensure the particles can pass through a typical cartridge filter. Currently, the engineering design standard of care in pretreatment of brackish water RO desalination and groundwater NF pre-treatment is the use of cartridge filters that typically possess 5 µm nominal pore size. The nanoparticles used in this work are on the order of 0.2 µm or less and would pass through a standard cartridge filter. Nanoparticle concentrations were determined after several trials until a flux decline was observed. Prior to each experiment, the nanoparticles were dissolved in DI water and the resultant suspension was sonicated in a water bath ultrasonicator for 30 min to maintain suspension.

### 2.2. Membrane Performance Testing

**Figure 1:**
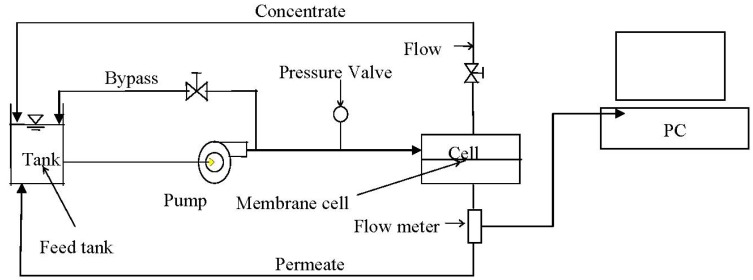
Flat sheet unit testing flow diagram.

Membrane productivity tests were performed using a CF042 cross flow flat sheet membrane filtration unit (CF042, Sterlitech, Kent, WA, USA). The membrane cell allows for evaluation of membrane film with an active surface area of 42 cm^2^. The cell dimension is 9.207 cm × 4.572 cm × 30 mL. The pre-cut membrane was loaded into the cell and the system was run under recommended pressure for 20 min with DI water to remove any residual chemicals from manufacturing. The water was then drained and the system was filled with testing solution. The schematic flow diagram for the flat sheet testing instrument is shown in Figure 1. A 1.5 gal reservoir provided feed water into a high pressure pump. The flow rate and pressure were adjusted by the two valves located on the bypass and concentrate flow tubes. The feed flow was maintained at 757 mL/min, providing a cross flow velocity of 0.18 m/s (Reynolds number is 307). The permeate and concentrate flows were recycled into the feed tank to ensure a constant background electrolyte condition. The temperature was maintained at 21 °C with a coil immersed in the feed tank and connected to a chiller unit. After a constant flux was achieved, an appropriate volume of premixed NaCl solution was added to provide a 0.05 M salt concentration. After the NaCl solution was added, the unit was allowed to equilibrate for 20 h to allow compaction of the new membranes. A dose of the resultant nanoparticle suspension was then added into the feed tank to provide a feed concentration of either 135 mg/L or 405 mg/L. The flux was monitored by a flow meter continuously for the duration of experiment and recorded on a laboratory computer.

For water qualities, pH, conductivity and the turbidity were measured at the beginning, the end, and several points during the experiments to maintain constant physical and chemical conditions throughout the test. Three runs were conducted for each membrane: baseline, 135 mg/L nanoparticle addition, and 405 mg/L nanoparticle addition. Each individual run lasted approximately twenty hours. The membrane productivities tested with different nanoparticles were studied in terms of flux decline and salt rejection over time. Relationships between surface properties and membrane productivity were quantitatively evaluated in this investigation.

### 2.3. Membrane Surface Properties

Membrane hydrophobicity and surface roughness were determined and evaluated in this study. Membrane hydrophobicity was determined by measuring the contact angle between the membrane surface and a water droplet. Contact angle measurements were obtained through the captive bubble technique. A goniometer manufactured by Rame-Hart was used to measure the contact angle.

The surface roughness of four different membranes was measured by a Digital Instruments Nanoscope Atomic Force Microscope. The AFM scans the surface with a cantilevered tip, generating a three-dimensional elevation map. The tip was operated in “tapping” mode to reduce the sample damage and maximize resolution. Surface elevation data can be used to determine the average roughness and the root mean squared (RMS) roughness. The average roughness is simply the average deviation of the peaks and valleys from the center plane; the RMS roughness is defined as the standard deviation of the peaks and valleys from the center plane. Both these parameters were used to determine the correlations between the fouling data and the membrane surface morphology.

## 3. Results

This section discussed the effect of chemical and physical characteristics of the membranes and nanoparticles on membrane performance. Theoretical analysis of the results was elaborated to understand the mechanisms of interaction between these nanoparticles and membrane productivity. The effect of nanoparticle concentration on the productivity of RO and NF membranes at a constant feed ionic strength are shown in the following figures. Results are presented in terms of relative flux as function of time. Relative flux is expressed as the flux at any time during the test divided by the initial flux (f/f_0_). The baseline represents the runs with the background solution (0.05 M NaCl) and without nanoparticles. The difference between the permeate flux with nanoparticles in the feed stream and the baseline indicates the net contribution of nanoparticles to membrane productivity. 

### 3.1. Effect of SiO_2_ on Flux Decline

**Figure 2:**
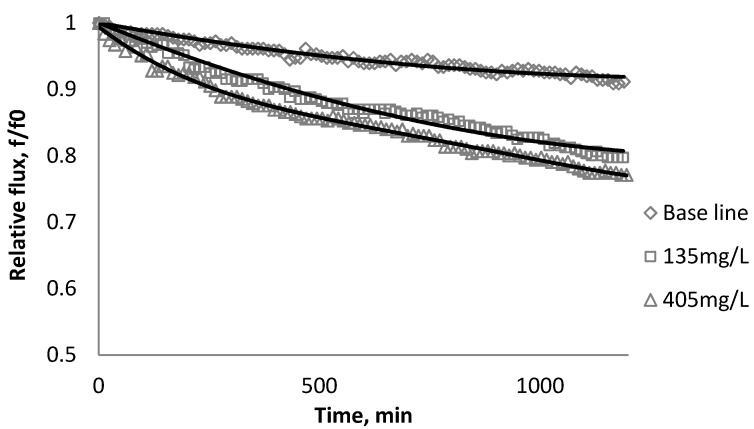
Relative flux as a function of time with SiO_2_ at three different particle concentrations for the BW30 membranes.

**Figure 3:**
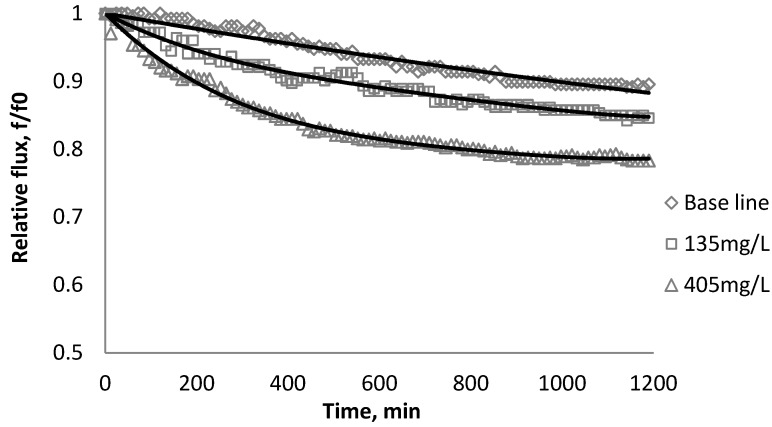
Relative flux as a function of time with SiO_2_ at three different particle concentrations for the XLE membranes.

**Figure 4:**
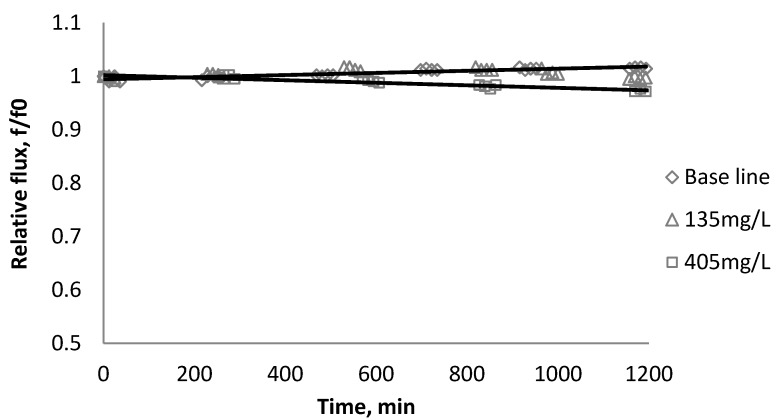
Relative flux as a function of time with SiO_2_ at three different particle concentrations for the CK membranes.

**Figure 5:**
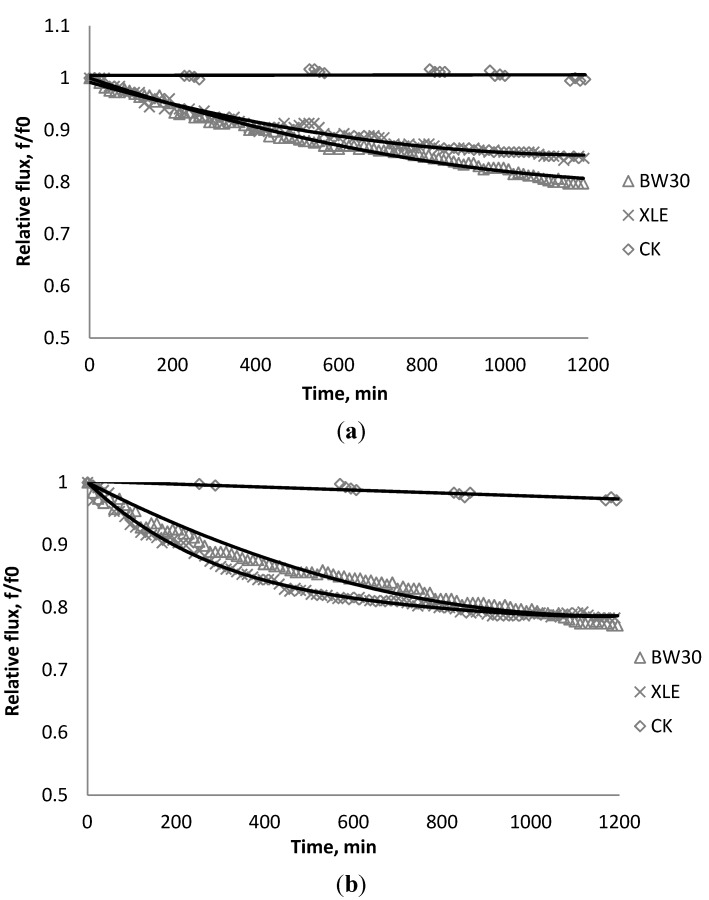
Relative flux as a function of time with: (a) 135 mg/L; (b) 405 mg/L as SiO_2_ in the feed stream to three different membranes.

Figure 2, Figure 3, Figure 4 show the effect of SiO_2_ concentration on the flux decline rate of RO and NF membranes at a constant ionic strength in the feed solution. The results from the BW30 and XLE membranes show that greater flux decline is obtained at a higher SiO_2_ particle dosage, while no obvious flux decline was observed for the CK membranes. With an increasing particle concentration, the rate of mass transport of particles toward the membrane surface increases, thereby, the overall rate of particle deposition onto the membrane surface increases. As a result, the total mass of deposited particles increases, which resulting in higher resistance to water permeating the membrane and thus reduced water flux. Figure 5 compares the rate of flux decline with a given concentration. The BW30 and XLE membranes have a higher relative flux decline rate, while the flux through the CK membrane decreases at a much lower rate relative to the other three. This behavior can be attributed to the pore sizes and to the difference in the surface roughness of these four membranes. RO membranes are almost nonporous while the nominal pore dimension for NF membranes is about 0.001 μm [18]. It is also noted that initial flux, shown in Table 3, also plays a role in determining the flux decline rate. Typically a higher initial flux results in a higher flux decline rate [7]. The initial flux for the BW30 membrane is slightly higher than the XLE membrane, but the flux decline rate is similar for these two membranes due to the differences in their surface morphology. The analysis of membrane surface roughness is discussed later in this study.

**Table 3:** Initial flux for membranes being tested using SiO_2_.

Initial flux	BW30	XLE	CK
m/s	1.31 × 10^−5^	1.00 × 10^−5^	6.54 × 10^−6^
gal/sfd	27.78	21.20	13.87

### 3.2. Effect of TiO_2_ on Flux Decline

**Figure 6:**
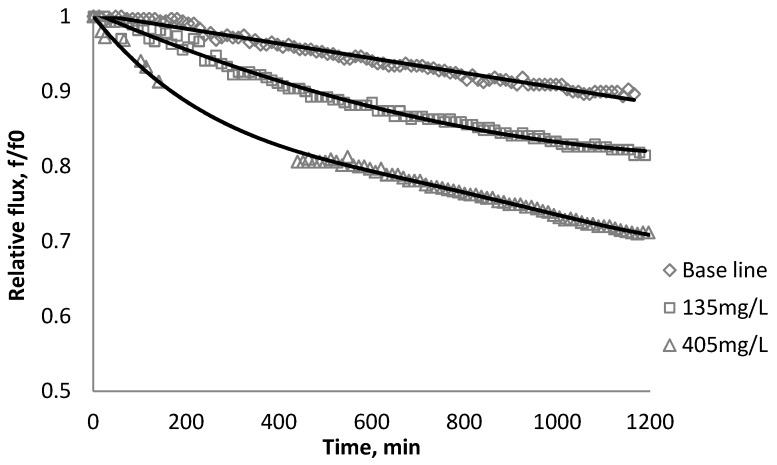
Relative flux as a function of time with TiO_2_ at three different particle concentrations for the BW30 membranes.

**Figure 7:**
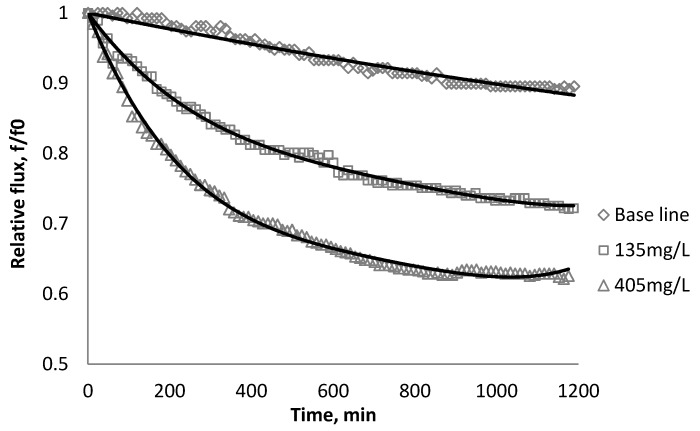
Relative flux as a function of time with TiO_2_ at three different particle concentrations for the XLE membranes.

**Figure 8:**
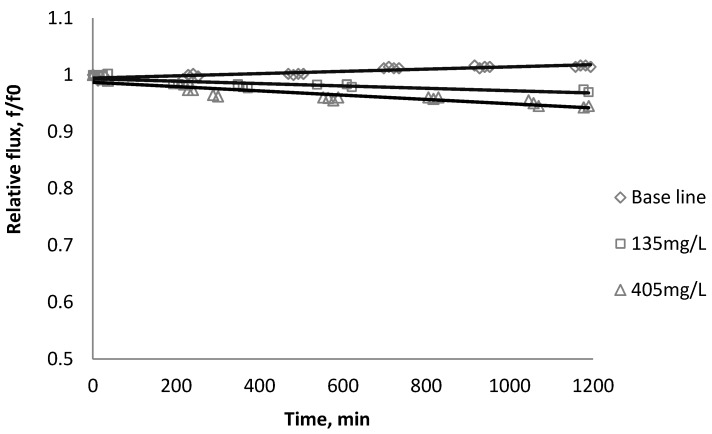
Relative flux as a function of time with TiO_2_ at three different particle concentrations for the CK membranes.

**Figure 9:**
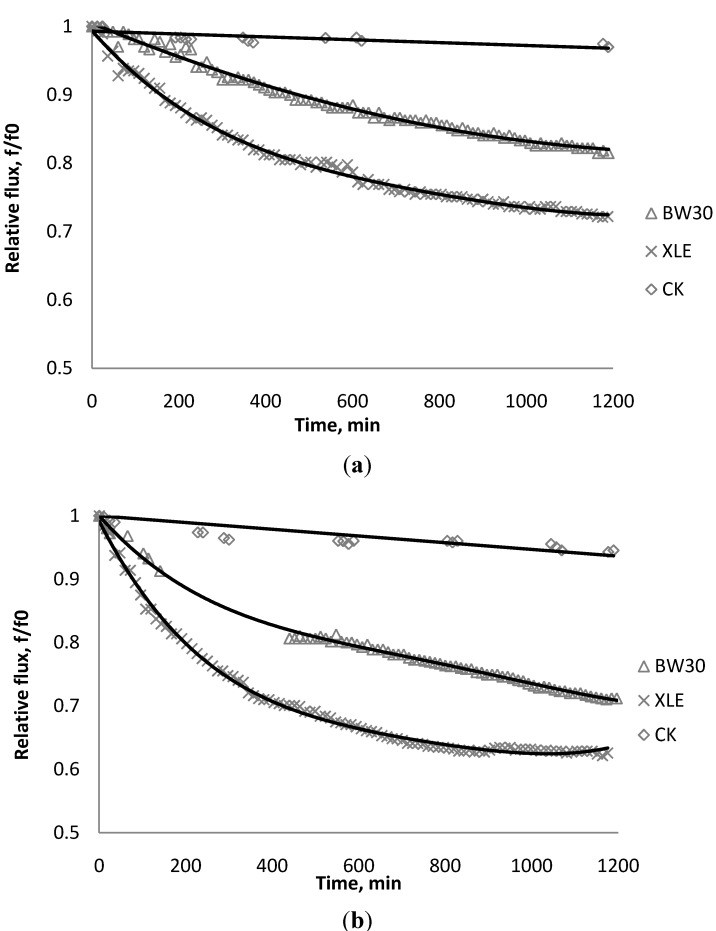
Relative flux as a function of time with: (a) 135 mg/L; (b) 405 mg/L as TiO_2_ in the feed stream to three different membranes.

The productivity of the RO and NF membranes with TiO_2_ is presented in Figure 6, Figure 7, Figure 8. Comparisons of the effect of TiO_2_ on flux decline rate with a given concentration are shown in Figure 9. The experiments were conducted with 0.05 M NaCl serving as a background solution. The feed flow was maintained at 757 mL/min. The initial flux is shown in Table 4. 

Results from Figure 6, Figure 7 indicate there is significant flux decline for the BW30 and XLE membranes, while Figure 9 shows no obvious flux decline for the CK membranes. When compared on the basis of percent flux decline at the end of each run, the membranes rank in the following order: *CK* < *BW30* < *XLE*. It is noted that compared with SiO_2_, TiO_2_ aggravates fouling for the BW30 and XLE membranes but does not affect the fouling rate for the CK membranes. One possible explanation is that the average size of TiO_2_ (15 nm) is smaller than SiO_2_ (80 nm) and the density of TiO_2_ (3.9 g/cm^3^) is higher than SiO_2_ (2.2 g/cm^3^), thus the cake layer formed from deposited TiO_2_ is less porous than that from SiO_2_, and thus produces higher resistance to water permeating the membranes.

**Table 4:** Initial flux for membranes being tested using TiO_2_.

Initial flux	BW30	XLE	CK
m/s	1.07 × 10^–5^	1.10 × 10^−5^	6.59 × 10^–6^
gal/sfd	22.69	23.33	13.98

### 3.3. Effect of CeO_2_ on Flux Decline

**Figure 10:**
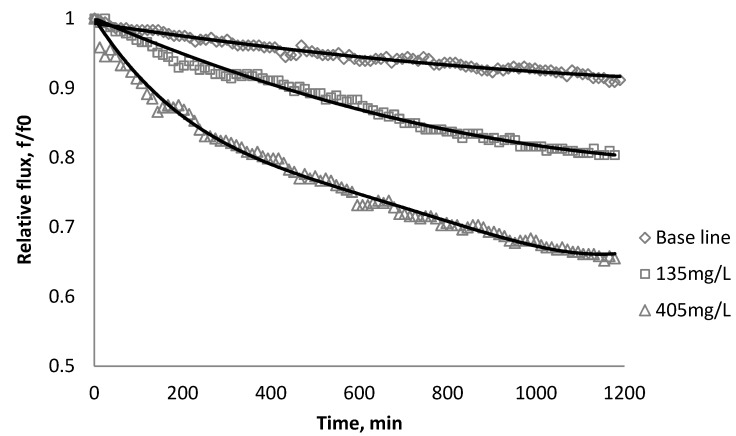
Relative flux as a function of time with CeO_2_ at three different particle concentrations for the BW30 membranes.

**Figure 11:**
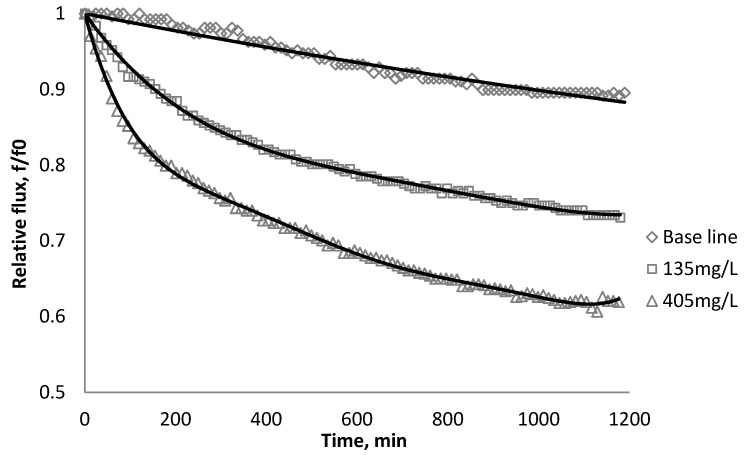
Relative flux as a function of time with CeO_2_ at three different particle concentrations for the XLE membranes.

**Figure 12:**
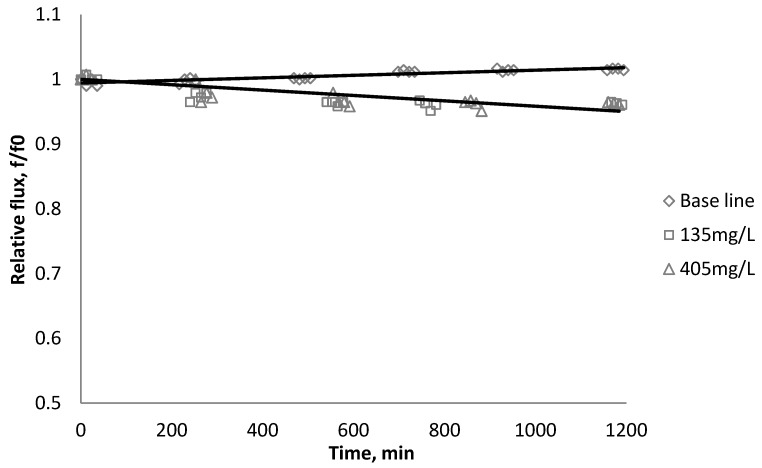
Relative flux as a function of time with CeO_2_ at three different particle concentrations for the CK membranes.

**Figure 13:**
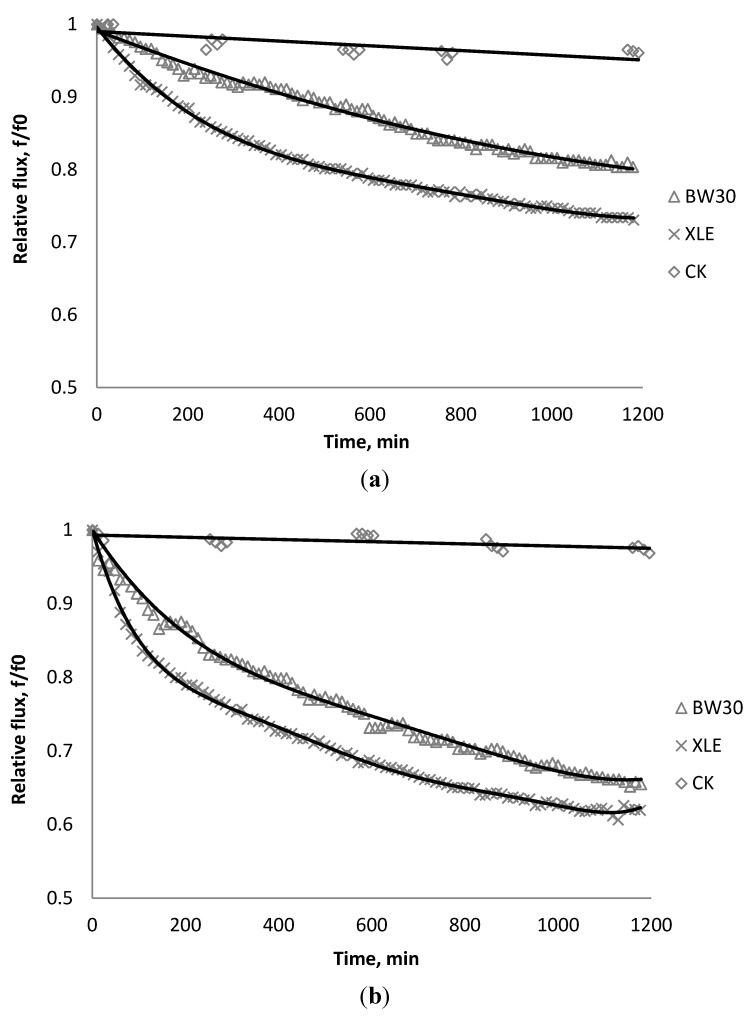
Relative flux as a function of time with: (**a**) 135 mg/L; (**b**) 405 mg/L as CeO_2_ in the feed stream to three different membranes.

Figure 10, Figure 11, Figure 12 show the effect of CeO_2_ on the productivity of BW30, XLE, and CA membranes at a constant ionic strength in the feed solution. Comparisons of the effect of CeO_2_ with a given concentration on flux decline rate are shown in Figure 13. 

The experiments were conducted with 0.05 M NaCl serving as a background solution. The feed flow was maintained at 757 mL/min. The initial flux is shown in Table 5. Similar to SiO_2_ and TiO_2_, there is significant flux decline for BW30 and XLE membranes when dosing with CeO_2_, while no obvious flux decline was observed for CK membranes. The magnitude of flux decline follows the same trend as testing with TiO_2_: the XLE membrane shows the most severe flux decline over the testing period, followed by the BW30 membrane; the CK membranes exhibit the least flux decline which indicates fouling resistant properties.

**Table 5:** Initial flux for membranes being tested using CeO_2_.

Initial flux	BW30	XLE	CK
m/s	1.29 × 10^−5^	1.24 × 10^−5^	6.68 × 10^−6^
gal/sfd	27.35	26.29	14.16

### 3.4. Effect of Cross Flow Velocity

**Figure 14:**
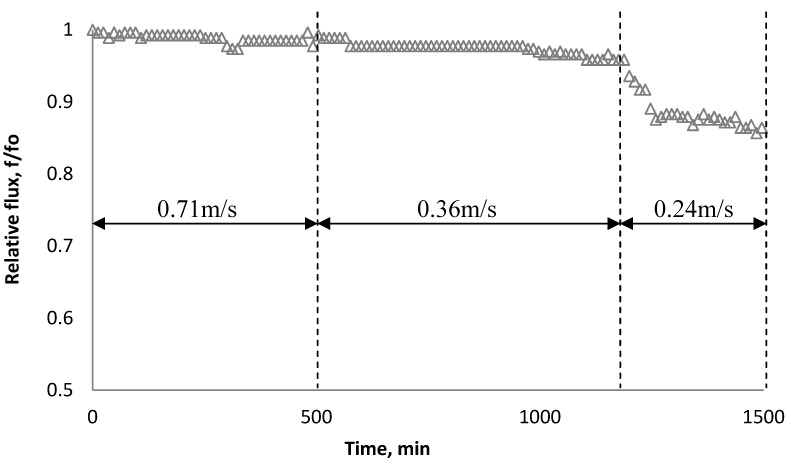
Relative flux as a function of time with the BW30 RO membrane at different cross flow velocity. The experiments were conducted with 135 mg/L SiO_2_ and 0.05 M NaCl serving as a background solution.

Experiments similar to those in Figure 14 were carried out with a 135 mg/L SiO_2_ particle suspension and the BW30 RO membrane to investigate the effect of cross flow velocity on the rate of flux decline. Results in Figure 14 indicate decreasing cross flow velocity from 0.71 m/s to 0.24 m/s (corresponding to Reynolds numbers of 1210 and 410, respectively) at an ionic strength of 0.05 M NaCl resulted in significant fouling.

### 3.5. Effect of Nanoparticles on Salt Rejection

**Figure 15:**
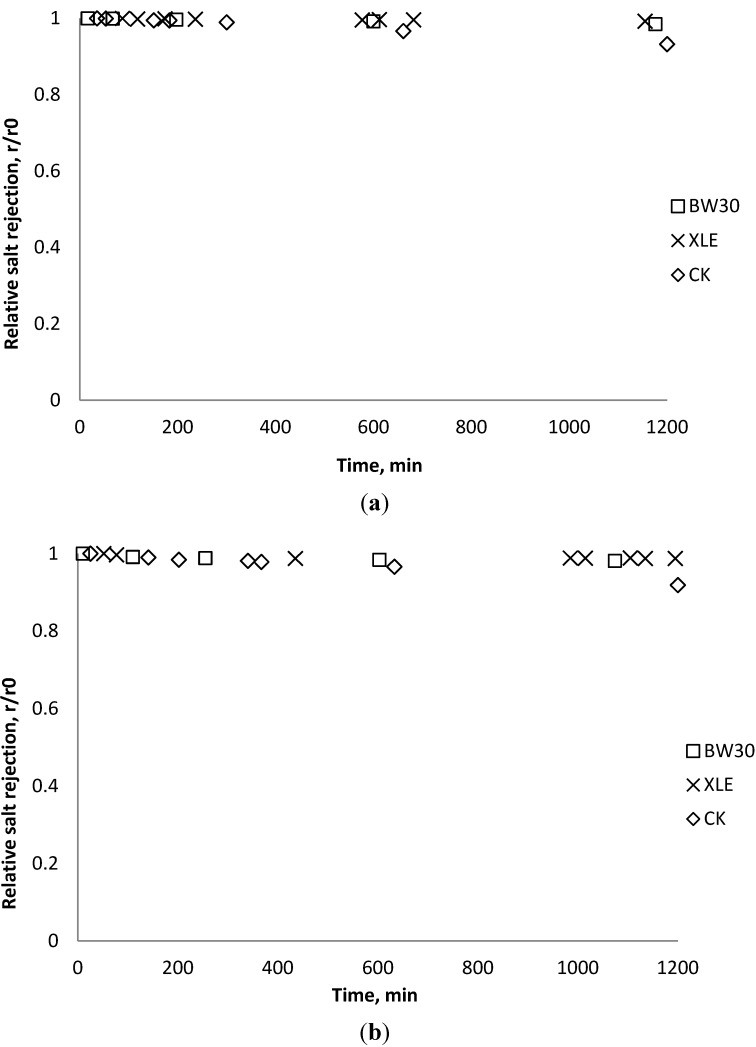
Relative salt rejection as a function of time with SiO_2_: (**a**) 135 mg/L; (**b**) 405 mg/L.

**Figure 16:**
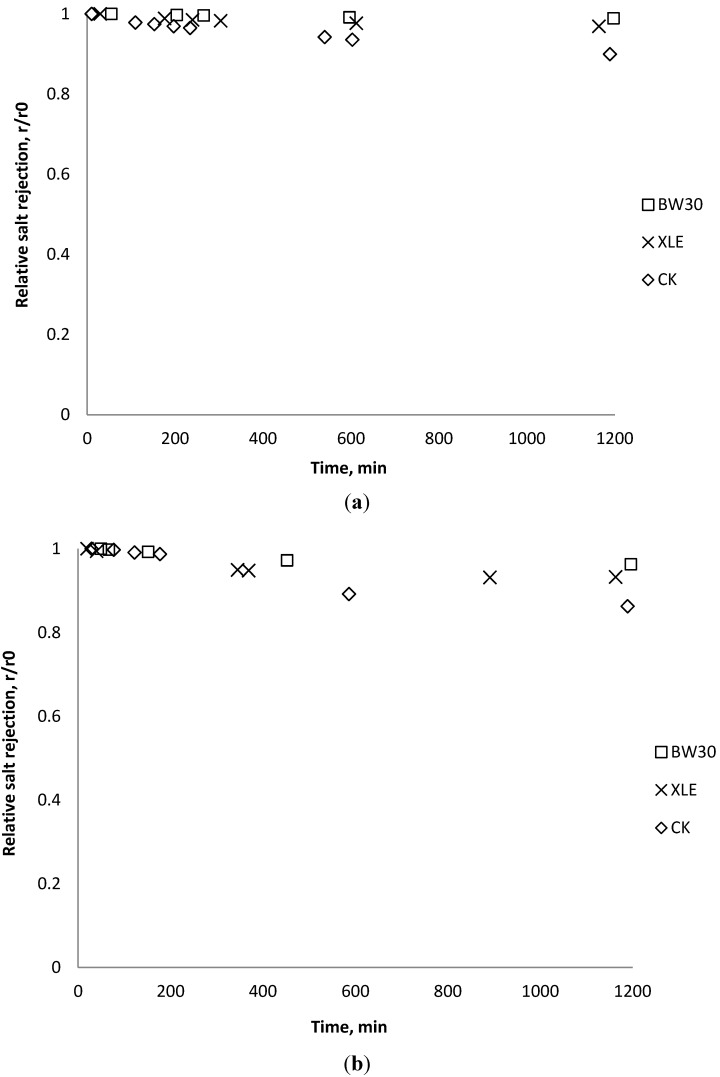
Relative salt rejection as a function of time with TiO_2_: (**a**) 135 mg/L; (**b**) 405 mg/L.

**Figure 17:**
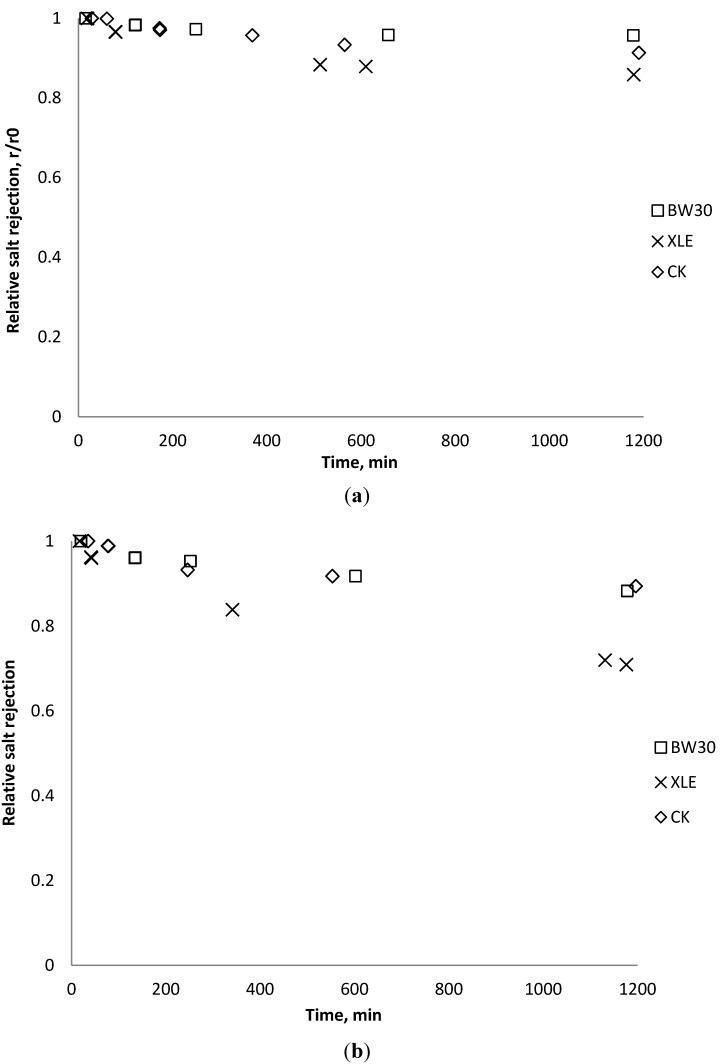
Relative salt rejection as a function of time with CeO_2_: (**a**) 135 mg/L; (**b**) 405 mg/L.

Salt rejection was measured at the beginning, the end, and several points during the fouling experiments. The results in Figure 15, Figure 16, Figure 17 show that for the BW30 membranes, salt rejection stays stable when tested with SiO_2_ or TiO_2_, but declines slightly when tested with CeO_2_. Similar to the BW30 membranes, the XLE membranes show constant salt rejection over time when tested with SiO_2_ or TiO_2_, but the rejection declines faster when the feed is injected with CeO_2_. For the three particles being tested, the CK membranes show a 10% decrease in salt rejection over the testing period. The decline in salt rejection can be attributed to the accumulated mass on the membrane surface, which may entrap dissolved salts, thus enhancing their passage through the membrane [19].

### 3.6. Fouling Experiment with Desalination Plant Water

Extended membrane productivity experiments were conducted using RO feed water from a sea water desalination plant located in Tampa Bay, FL, USA. This plant is capable of producing 25 MGD of desalinated water. The tested water was sampled after the cartridge filters but before the RO feed pressure pump. The feed water TOC level in pretreated seawater approximated 4.2 mg/L, the salinity ranged from 43.8 to 44.6, and the pH measured approximately 6.8 pH units.

**Figure 18:**
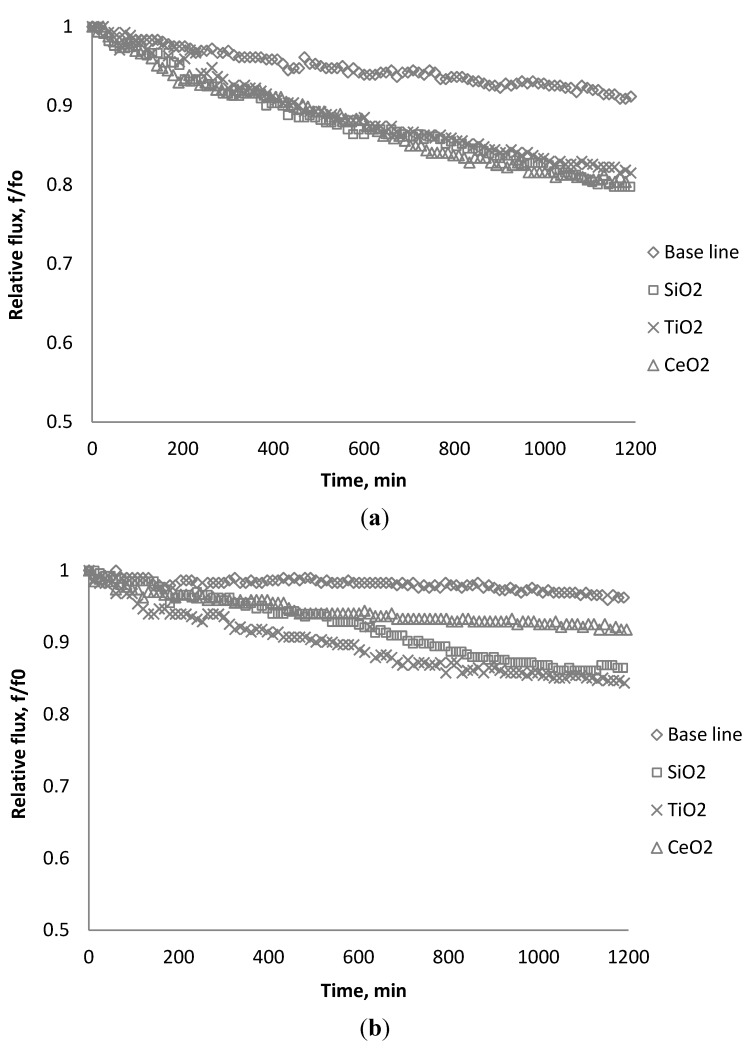
Relative flux as a function of time using different source water: (**a**) 0.05 M NaCl; (**b**) diluted reverse osmosis (RO) feed water from Tampa Bay desalination plant.

Before the fouling experiment, the raw water was diluted ten to one to reduce the salt concentration such that the feed water conductivity was about 4800 μs/cm, which is similar to the laboratory tested condition previously examined. Each particle was dosed into the feed tank with a concentration of 135 mg/L after 20 h of particle free solution testing. The fouling behavior of the BW30 membranes were investigated and the relative flux with each particle addition using 0.05 M NaCl solution and diluted Tampa Bay water are shown in Figure 18a,b. Using a 0.05 M NaCl solution as the basis for the suspensions, the flux decreases at a similar speed. When using RO feed water from the Tampa Bay desalination plant, the permeate flux declines at different rates; feeding with TiO_2_ results in the highest flux decline, and CeO_2_ appears to relieve the fouling compared to other two particles. It is noted that the flux decline between the laboratory-grade and diluted seawater feed solutions were found to be different. The scope of the research presented herein did not fully evaluate the constituents present that would explain this difference; however, it is noted that the ionic strength differences may help explain the trends observed. Additional studies are being conducted to further elucidate observed differences.

### 3.7. Correlation of Membrane Surface Properties with Membrane Productivity

In this section, membrane surface properties (contact angle and surface morphology) are investigated. The flux decline rate is shown to be related to these physical and chemical properties of membrane surface.

#### 3.7.1. Surface Morphology

**Figure 19:**
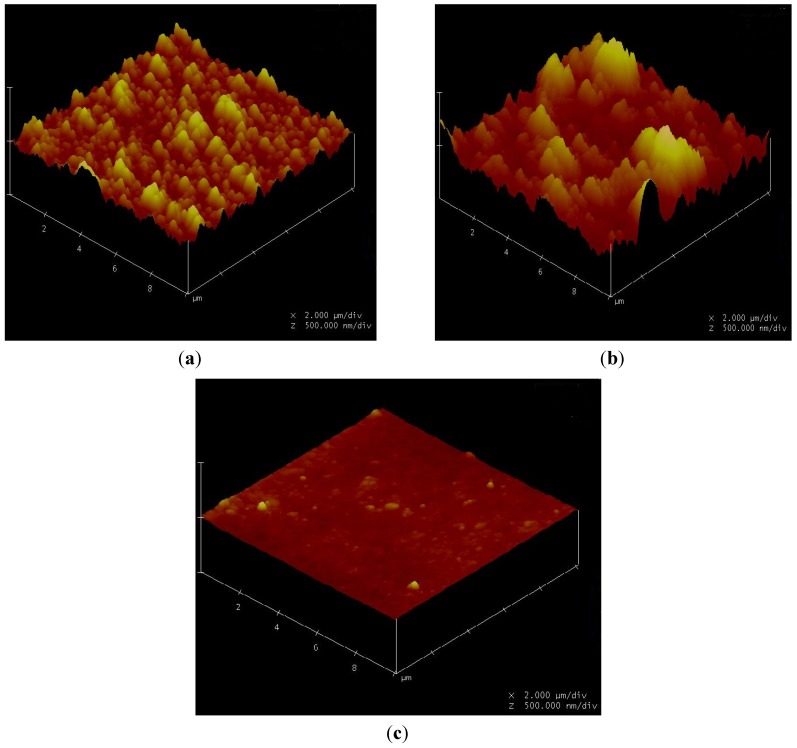
Atomic force microscopy (AFM) images of (**a**) DOW BW30 (RO); (**b**) DOW XLE (RO); (**c**) GE Osmonics CK (NF). Note the X and Y dimensions are both 10 μm (2 μm/div), and the Z scale us 1 μm (500 nm/div).

Membrane surface morphologies were measured using an AFM and are shown in Figure 19. The BW30 and XLE membranes depict a ridge-and-valley morphology, while the CK membranes show a smoother surface. The parameters obtained from AFM analysis are shown in Table 6. By comparing with the flux decline rate in Figure 5, Figure 9, Figure 13, it can be concluded for the particles being tested, membranes with a higher mean roughness or root mean square (RMS) roughness suffer from flux decline at a higher speed, while membranes with smoother surfaces result in less flux decline. The mean value of the membrane surface was found to have no correlation to membrane productivity.

**Table 6:** AFM analysis of surface roughness.

Membrane	Average roughness (nm)	RMS * (nm)	Mean (nm)
BW30	30	38.1	0
XLE	81.1	105.8	0.123
CK	5.1	6.6	0.018

* RMS: root mean square.

#### 3.7.2. Contact Angle

Contact angles of clean membranes and membranes with particle deposition were measured and are shown in Table 7. There is no correlation between contact angle and flux decline rate. For each type of membranes, SiO_2_ and CeO_2_ increase the surface hydrophilicity, while TiO_2_ increases the surface hydrophilicity of the BW30 and XLE membranes and decreases the hydrophilicity of the CK membranes.

**Table 7:** Contact angle of clean and particle deposited membranes.

Membrane	Condition	Contact angle
BW30	Clean	58.4
	SiO_2_	48.8
	TiO_2_	54.0
	CeO_2_	51.5
XLE	Clean	61.5
	SiO_2_	54.2
	TiO_2_	53.2
	CeO_2_	46.4
CK	Clean	61.1
	SiO_2_	58.8
	TiO_2_	62.9
	CeO_2_	54.5

## 4. Simulation of Cake Deposit Membrane Processes

### 4.1. Model Development

For pressure-driven membrane processes, permeate water flux can be expressed by Equation (3), where the mass transfer coefficient *k_wi_* is a function of membrane thickness and described in Equation (4):

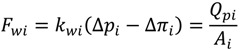
(3)

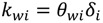
(4)
where *θ_wi_* is an empirical coefficient that correlates the mass transfer coefficient to the localized membrane thickness *δ_i_*. In the cake growth process, *δ_i_* equals to the sum of clean membrane thickness (*δ_m_*) and the cake thickness (*δ_c_*).

The cake thickness, *δ_c_*, is a key unknown for the prediction of permeate flux during the experiment. The rate of cake layer growth is given by [25]:

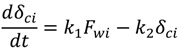
(5)
where *k_1_* and *k_2_* are constants. Equation (5) is based on the assumptions that the cake growth is proportional to the permeate flux and the particle back diffusion due to shear stresses increases by the membrane channel constriction as the cake grows. At the early stages of the experiment, when the membrane channel is not constricted by the cake grown and shear force is minimal. Equation (5) can be simplified as:

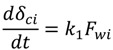
(6)

The membrane channel is discretized to 500 uniform slices as described in the work of Fang and Duranceau [26]. The localized permeate flux can be determined by Equation (7):

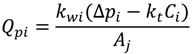
(7)

With consideration of pressure drop along the membrane channel, the localized trans-membrane pressure is determined by Equations (8) and (9):

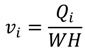
(8)
where *W* is the membrane element width and *H* is the channel height. As feed flow travels in the membrane channel, transmembrane pressure decreases due the hydraulic head loss. The transmembrane pressure profile in the membrane channel can be described by Equation (9) [27]:

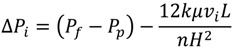
(9)
where *P_f_* and *P_p_* is the feed and permeate pressure, *k* is the friction coefficient and is determined in the work of Fang and Duranceau [12], *µ* is the fluid viscosity, *L* is the membrane channel length, and *n* is the number of uniform slices for channel. Substituting Equation (7) into Equation (5), the cake thickness *δ_ci_* at each time increment *t_i_* can be determined assuming initial thickness equals to zero. Because the localized *k_wi_* is affected by the localized thickness *δ_i_*, which is affected by the surface roughness. The cake thickness *δ_ci_* is a localized variable determined by the permeate flux and surface roughness. The discretized forms of Equations (5) and (6) are:

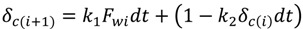
(10)

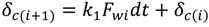
(11)

### 4.2. Numerical Representation of Membrane Surface Morphology

With the surface parameters given in Table 5, the hypothetical clean membrane surfaces can be generated by MATLAB using the NORMRND and SMOOTHN functions [26]. Then the initial localized *k_wi_* and *F_wi_* can be calculated by Equations (3) and (4). Assuming the time interval *dt* is 12 min, the localized cake layer growth can be calculated at each time increment described by Equations (10) and (11). The overall cake thickness is the average of the cake thickness at each uniform slice. Two cases are considered depending on whether or not the back transport is significant.

### 4.3. Effect of Surface Roughness on Overall Cake Growth Rate

At an early stage of the experiment, when particle back transport can be negligible (*k_1_ F_wi_ ≫ k_2_ δ_ci_*), the permeate flux is controlled by the resistance of cake growth. The permeate flux decreases linearly due to the quick growth of cake. This condition is under the assumption that the rate of cake growth is much higher than the rate of particle back diffusion. By fitting the experimental data into Equation (11) using trial and error, *k*_1_ can be solved. For different membranes tested with different particles, this condition only held until certain percentage of flux decline. Table 8 shows the percentage of flux decline when the back diffusion can be negligible.

**Table 8:** Percentage of flux decline when *k_1_ F_wi_ ≫ k_2_ δ_ci_* condition held.

Membrane	SiO_2_	TiO_2_	CeO_2_
BW30	93%	86%	92%
XLE	90%	80%	90%
CK	N/A	N/A	97%

At the next stage of the experiment, the particle back diffusion term (*k_2_ δ_ci_)* gains importance in comparison with the cake growth term (*k_1_ F_wi_)* in Equation (5). The permeate flux starts to decline at a lower rate compared with the previous stage. *k_2_* was also determined by fitting the simulated permeate flux to the experimental monitored flux data using trial and error. The ratio of *k_1_* over *k_2_* are used to evaluate the significance of the cake growth term (*k_1_ F_wi_*) and particle back diffusion term (*k_2_ δ_ci_)* which is shown in Table 9.

**Table 9:** *k*_1_ and *k*_2_ values for different membranes tested with different particles.

Membrane	Particle	Cake growth term	Particle back diffusion term	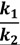	RMS	Applied Pressure, psi
		*k*_1_	*k*_2_			
XLE	CeO_2_	0.105	0.00055	191	105.8	130
BW30		0.028	0.00012	233	38.1	260
CK		0.015	0.00009	167	6.6	230
XLE	SiO_2_	0.029	0.00012	236	105.8	130
BW30		0.020	0.00007	286	38.1	260
CK		no flux decline, 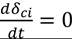		0	6.6	230
XLE	TiO_2_	0.060	0.00025	240	105.8	130
BW30		0.023	0.00008	295	38.1	260
CK		no flux decline, 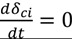		0	6.6	230

In a cake resistance model, a smaller ratio of 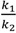 indicates back diffusion *k_2_* is more significant and more particles remain in the bulk flow. The XLE and BW30 membranes have a higher 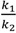 value than the CK membranes, indicating the permeate flux is determined by the cake layer resistance and back diffusion is less significant. There is no observed flux decline using the CK membranes with SiO_2_ or TiO_2_ deposition, so Equation (5) can be approximated to zero and 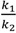 is determined by the following:

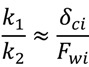
(12)
where *δ_ci_* equals zero and *k_2_ → ∞*. It is also noted that in most cases the XLE and BW30 membranes have a higher RMS value than the CK membranes, which corresponds to a higher 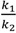 ratio. In addition, when comparing the XLE and BW30 membranes, a higher applied pressure tends to produce more resistance for particle back diffusion, thereby the BW30 membranes have a higher 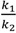 ratio than the XLE membranes.

### 4.4. Effect of Non-Homogeneous Surface on Particle Deposition Distribution

**Figure 20:**
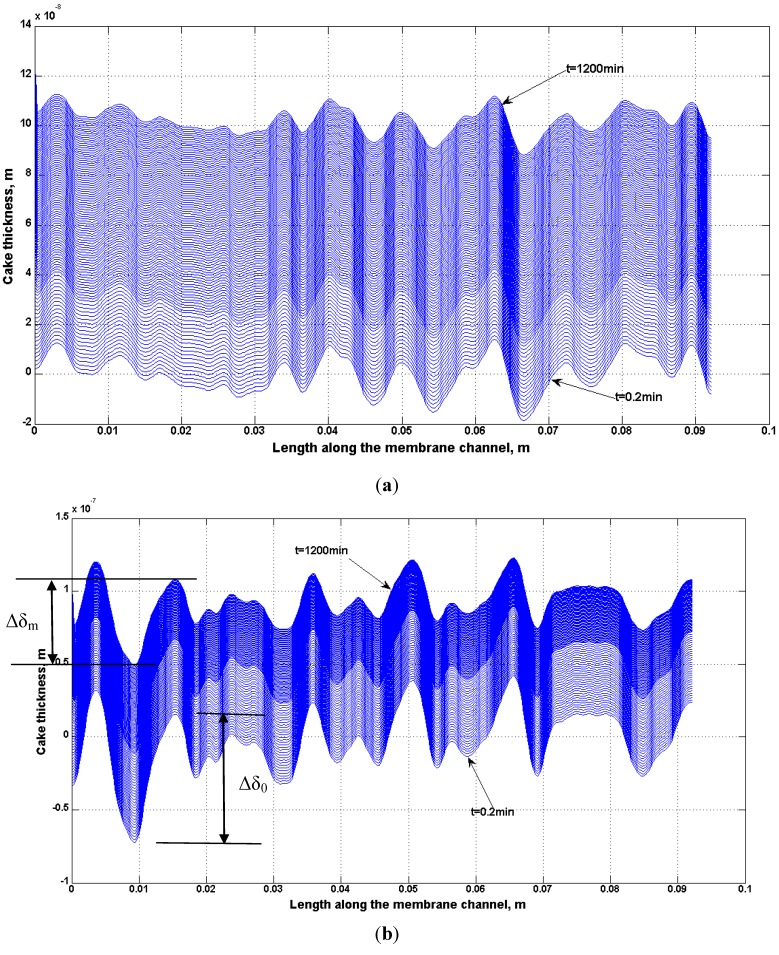
Simulation of cake thickness growth along the membrane channel during the experiments: (**a**) BW30 RO; (**b**) XLE RO. Noted that feed stream contained 135 mg/L SiO_2_ and ionic strength was maintained as 0.05 M NaCl.

A non-uniform permeability of membranes has been simulated using Equation (4). The growth of the deposit particles on the BW30 and XLE membranes has been studied. Using the empirical coefficients shown in Table 9, the localized cake thickness can be calculated by Equations (10) and (11). As an example, Figure 20 presents the cake thickness distribution through spatial and temporal variation for the BW30 and XLE membranes. At the beginning of the experiment, the distribution of cake thickness along the membrane channel is similar to the ridge-and-valley distribution on the clean membrane surface. By comparing the cake thickness distribution at the beginning and at the end of the experiment, the magnitude between ridge and valley is gradually diminished. This difference is demonstrated in Figure 20b in terms of Δδ_0_ as the cake thickness difference between the ridge and valley at the beginning and Δδ_m_ as in the end of the experiment. This observation indicates that the valley areas of the membranes are filled up by particles in a higher rate than the ridges which is one of the primary reasons for flux decline.

### 4.5. Comparison of Simulation and Experimental Results

**Figure 21:**
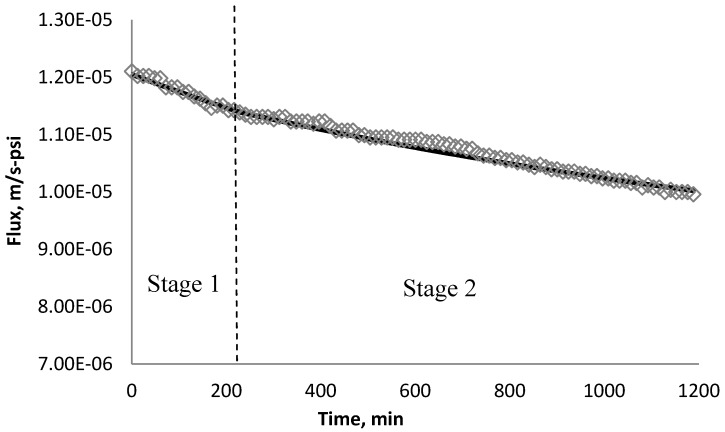
Permeate flux over time with 135 mg/L SiO_2_ in the feed stream tested on BW30 membrane.

**Figure 22:**
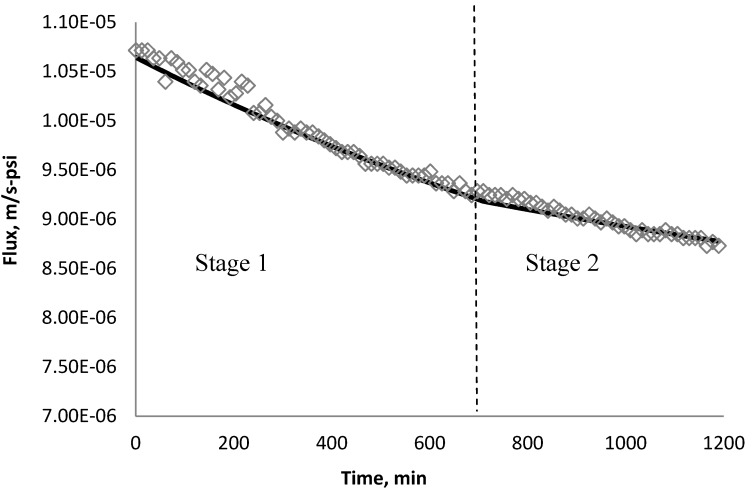
Permeate flux over time with 135 mg/L TiO_2_ in the feed stream tested on BW30 membrane.

**Figure 23:**
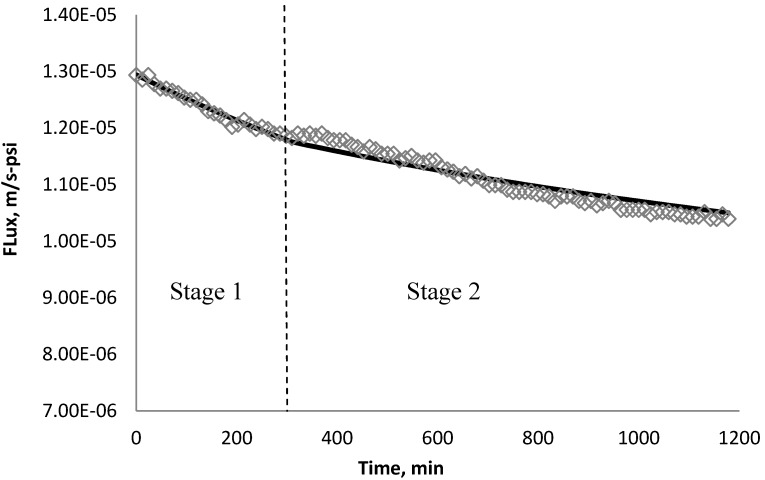
Permeate flux over time with 135 mg/L CeO_2_ in the feed stream tested on BW30 membrane.

**Figure 24:**
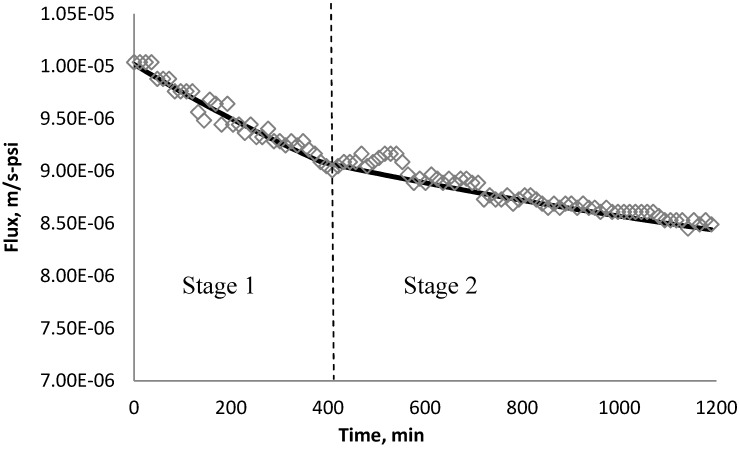
Permeate flux over time with 135 mg/L SiO_2_ in the feed stream tested on XLE membrane.

**Figure 25:**
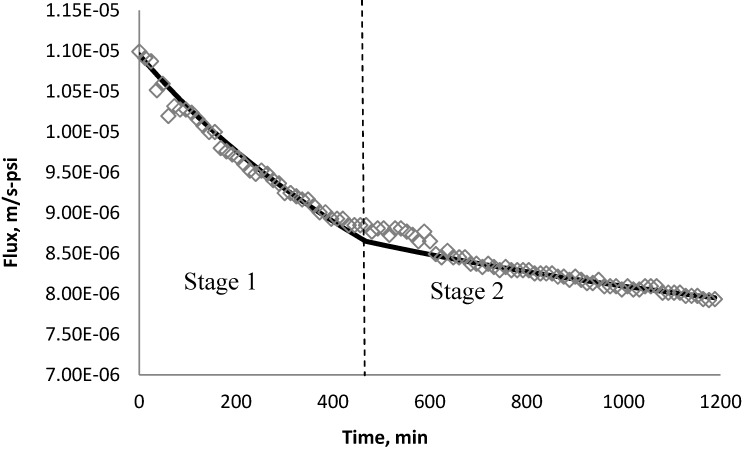
Permeate flux over time with 135 mg/L TiO_2_ in the feed stream tested on XLE membrane.

**Figure 26:**
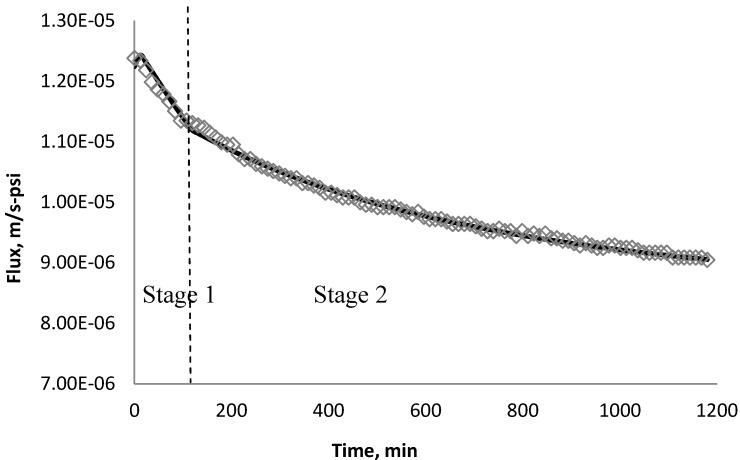
Permeate flux over time with 135 mg/L CeO_2_ in the feed stream tested on XLE membrane.

Using *k_1_* and *k_2_* from Table 9, the fitted lines and the experimental data for BW30 and XLE membranes are plotted from Figure 21, Figure 22, Figure 23, Figure 24, Figure 25, Figure 26. The solid line represents the simulation fit and the markers are the monitored data. In the first stage, the cake growth rate is proportional to the flux decline rate and the back diffusion is minimized. In the second stage, the cake growth rate decreases due to the back diffusion of particles and the flux curve flattens slightly. The fit lines appear to be in good agreement with the experimental data, indicating the estimated *k*_1_ and *k*_2_ values are valid.

## 5. Conclusions

In this work, bench scale membrane productivity experiments were conducted to investigate the role of membrane surface properties on the productivity of RO and NF. Three different nanoparticles were injected into the feed stream and the intensity of flux decline was characterized in terms of relative flux (f/f_0_). In the three cases evaluated, the intensity of flux decline was influenced by membrane surface morphology. The AFM analysis reveals that the higher flux decline rate of the XLE and BW30 membranes compared to that of the CK membranes is due to the inherent ridge-and-valley morphology of the XLE and BW30 membranes. This unique morphology increases the surface roughness, leading to particles accumulation in the valleys, and causing more flux decline than in smoother membranes. Simulation results also indicate back diffusion of deposit particles is more severe on the smooth membranes than on the rough membranes. The valley areas of membranes have the ability to capture the particles and protect them from back diffusion.

Extended productivity experiments were conducted using the BW30 membranes to compare the effect of different particles on diluted seawater *versus* laboratory controlled water. When supplying the membranes with laboratory controlled water, the flux decline at a similar rate regardless of particle type. But when supplying the membranes with a pretreated yet diluted seawater supply, CeO_2_ results the least flux decline compared to the other particles.

## Symbols and Abbreviations

RO: reverse osmosis
NF: nanofiltration
AFM: atomic force microscopy
TMP: trans-membrane pressure
RMS: root mean square
*n*: amount of uniform slice for channel discretization
Δ*p*: transmembrane pressure, psi
*P_f_*: feed pressure, psi
*P_c_*: concentrate pressure, psi
∆π,∆π^∗^_m_: osmotic pressure, psi
*Q_f_*: feed flow, m^3^/s
*Q_c_*: concentration flow, m^3^/s
*Q_pi_*: permeate flow, m^3^/s
*Q_i_*: flow in the membrane channel, m^3^/s
*k_m_*, *k_c_*, *k_p_*: resistance coefficient for membrane, cake layer, and pore constriction
*µ*: dynamic viscosity
*F_wi_*: solvent permeate flux, gal/sfd
*J_i_*: solute permeate flux, mg/sfd
*k_wi_*: solvent mass transfer coefficient, m/s-psi
*θ**_wi_*: empirical coefficient
*δ**_i_*: overall membrane thickness, m
*δ**_m_*: clean membrane thickness, m
*δ**_c_*: cake thickness, m
*k*_1_: cake growth term
*k*_2_: particle back diffusion term
*dt*: time interval, min
*W*: membrane channel width, m
*H*: membrane channel height, m
*L*: membrane channel length, m
*ρ*: density of water, kg/m^3^
*v_i_*: cross flow velocity, m/s
*k*: friction coefficient
